# The anti-inflammatory effect of *Andrographis paniculata* (Burm. f.) Nees on pelvic inflammatory disease in rats through down-regulation of the NF-κB pathway

**DOI:** 10.1186/s12906-016-1466-5

**Published:** 2016-11-25

**Authors:** Wei Zou, Zuoqi Xiao, Xiaoke Wen, Jieying Luo, Shuqiong Chen, Zeneng Cheng, Daxiong Xiang, Jian Hu, Jingyu He

**Affiliations:** 1 Key Laboratory of Hunan Province for Traditional Chinese Medicine in Obstetrics and Gynecology Research, Hunan Provincial Maternal and Child Health Care Hospital, No. 53 XiangChun Road, Changsha, 410008 China; 2 School of Pharmaceutical Sciences, Central South University, Changsha, 410011 China; 3 College of Pharmacy, Hunan University of Chinese Medicine, Changsha, 410007 China; 4 Clinic Pharmacy Research Laboratory, the Second Xiangya Hospital of Central South University, Changsha, 410011 China; 5 Guangzhou Institute of Advanced Technology, Chinese Academy of Sciences, No. 1121 Haibin Road, Guangzhou, 511458 Guangdong China

**Keywords:** *Andrographis paniculata* (Burm. f.) Nees, Anti-inflammatory, Pelvic inflammatory disease, NF-κB, Rats

## Abstract

**Background:**

*Andrographis paniculata* (Burm. f.) Nees (APN), a principal constituent of a famous traditional Chinese medicine Fukeqianjin tablet which is used for the treatment of pelvic inflammatory disease (PID), has been reported to have anti-inflammatory effect in vitro. However, whether it has pharmacological effect on PID in vivo is unclear. Therefore, the aim of this study is to test the anti-inflammatory effect of APN and illuminate a potential mechanism.

**Methods:**

Thirty-six female specific pathogen-free SD rats were randomly divided into control group, PID group, APN1 group, APN2 group, APN3 group and prednisone group. Pathogen-induced PID rats were constructed. The APN1, APN2 and APN3 group rats were orally administrated with APN extract at different levels. The prednisone group rats were administrated with prednisone. Eight days after the first infection, the histological examination of upper genital tract was carried out, and enzyme-linked immunosorbent assay (ELISA) was carried out using homogenate of the uterus and fallopian tube. Furthermore, immunohistochemical evaluations of NF-κB p65 and IκB-α in uterus was conducted.

**Results:**

APN obviously suppressed the infiltrations of neutrophils and lymphocytes, and it could significantly reduce the excessive production of cytokines and chemokines including IL-1β, IL-6, CXCL-1, MCP-1 and RANTES in a dose-dependent manner. Furthermore, APN could block the pathogen-induced activation of NF-κB pathway.

**Conclusion:**

APN showed potent anti-inflammatory effect on pathogen-induced PID in rats, with a potential mechanism of inhibiting the NF-κB signal pathway.

## Background

Pelvic inflammatory disease (PID) is a common gynecological disease that usually causes ectopic pregnancy, tubal factor infertility and chronic pelvic pain, which has been deemed as a great threat for life quality of woman. It includes endometritis, salpingitis, peritonitis, *etc*., whose etiopathogenisis is the infection of pathogenic microorganisms in upper genital tract [1]. Infiltrations of neutrophils and lymphocytes in the upper genital tract could be employed as a criteria to diagnose PID in clinic, which could be observed on hematoxylin and eosin (H & E)-stained biopsy sections [2]. With the inflammatory cell recruitment, amount of proinflammatory cytokines, such as interleukin (IL)-1β, IL-6, *etc*., are released excessively in local tissue and play an important role in the pathogenesis of PID [3]. The important reason for the excessive production and release of proinflammatory cytokines is the activation of nuclear factor-kappa B (NF-κB) signaling pathway when pathogens are recognized by their receptors [4].

According to the guidelines from the Center for Disease Control and Prevention (CDC) in US, antibiotics is the first choice for the treatment of PID [5], but the bacterial drug resistance and drug side effects are shortages for clinical use of antibiotics. Therefore, new complementary medicines used for PID are needed for further improvement in clinical outcomes. *Andrographis paniculata* (Burm. f.) Nees (APN) is a famous traditional medicine, widely used to treat sore throat, flu, and upper respiratory tract infections in many Asian countries [6]. Phytochemical studies on APN have found the principal bioactive compound andrographolide [7] and many other constituents, including diterpenoids, flavonoids, quinic acids, xanthones [8, 9], and noriridoids [10, 11]. Based on the abundant bioactive constituents, APN showed many salutary effects, such as anticancer [12], hepatoprotective [13], antiviral [14, 15], antipyretic and analgesic [16] effects, *etc*. Interestingly, the immunomodulatory [17], antioxidant [18], anti-inflammatory [19] and antimicrobial [20, 21] activities of APN were also reported, which may suggest the clinical use of APN in treatment for PID. Besides, APN is the principal constituent in a famous traditional Chinese medicine Fukeqianjin tablet which is used to treat PID. However, whether APN has pharmacological effect on PID has not been eliminated yet. In this study, we test the anti-inflammatory effect of APN on PID rats, and illustrate a potential mechanism of this activity.

## Methods

### Reagents and materials

Pentobarbital was from Xiya Reagent (Chengdu, China). Progesterone injection was obtained from Xianju Pharma (Taizhou, China). Distilled water was used in all of this experiment. Absorbable gelatin sponge was from Jinling Pharmaceutical Co. (Nanjing, China). The dried APN was purchased from Tianxiang Co. (Yueyang, China), which was identified by Prof. Zhuxin Wang (Hunan university of Chinese medicine, Changsha, China), with the total amount of andrographolide and dehydroandrographolide about 1%. A voucher specimen (No. CXL20150610) is deposited in the Key Laboratory of Hunan Province for Traditional Chinese Medicine in Obstetrics & Gynecology Research (Changsha, China). Prednisone acetate tablets were obtained from Guangdong Huanan Pharma (Guangdong, China). According to the previous method [22], a 1000 g of dried APN was extracted with 80% ethanol at room temperature and filtrated. The solution was freeze-dried to yield 62.5 g of the APN extract.

### Rat PID model construction and sample collection

The animal experimental procedure was approved by the Animal Care and Use Committee of Central South University. Thirty-six female specific pathogen-free SD rats, 9-week aged and weighing 220–240 g, were randomly divided into 6 groups, including control group, PID group, APN1 group, APN2 group, APN3 group and prednisone group. Rats were acclimated for 7 days and then injected subcutaneously with 10 mg progesterone. One week later, the PID model construction was carried out referring to our previous method with some revisions [23]. Absorbable gelatin sponge, a volume of 0.125 ml, was immersed in microbe-mixing solution with *U. urealyticum* (t-strain mycoplasma) concentration of 1 × 10^8^ ccu/ml and pathogenic *E. coli* concentration of 1 × 10^8^ cfu/ml. Each upper genital tract of all rats except control group rats was inserted with a microbe-containing gelatin sponge, and then the rat was forced to be down for 3 min. The microbe-free gelatin sponges were implanted into the cervixes of control group rats. Four times infections were conducted with a 2-day interval. From the first infection, the APN1, APN2 and APN3 group rats were orally administrated with APN extract at a dose of 167, 334 (clinical dose) and 668 mg/kg/day, respectively. The prednisone group rats were administrated with prednisone at a dose of 1.7 mg/kg. Eight days after the first infection, rats were injected subcutaneously with pentobarbital at a dose of 30 mg/kg. The right uterus and fallopian tube were collected and restored at −80 °C, and the left uterus and fallopian tube was immersed in neutral-buffered formalin (10%). At last, rats were sacrificed by cervical dislocation.

### Histological evaluation

After paraffin embedding, the left uterus and fallopian tube was cut into 2 μm sections, followed by staining with H & E. The semi-quantification was carried out according to the previous method [24]. Three parts of each slide (tissue) were checked under microscopy (×100) by a blinded observer. The inflammation of each uterus and fallopian tube was semi-scored by evaluation on the extent of inflammatory cells infiltration (graded from 0 to 3).

### Enzyme-linked immunosorbent assay (ELISA)

Each right uterus and fallopian tube was weighted, immersed in physiologic saline at the ratio of 5:1 (v/w), and homogenized. The amounts of IL-1β, IL-6, CXCL-1, MCP-1 and RANTES in homogenate were determined by using ELISA kits (Neobioscience, Beijing, China). The total protein in tissue homogenate was measured with bicinchonininc acid (BCA) protein assay kit (Beyotime, Shanghai, China), and concentrations of these cytokines and chemokines were expressed as μg/g protein of homogenate.

### Immunohistochemical evaluation

The paraffin embedded uterus was cut in to 2 μm sections, and then the paraffin was removed through xylene. The slide was boiled in 10 mM sodium citrate (pH 6.0) for 30 min for antigen retrieval. Non-specific binding sites were blocked by bovine serum. The specimens were incubated with NF-κB p65 or IκB-α primary antibody (Abclonal, Maryland, US) at 37 °C followed by secondary antibodies incubation. After that, the slices were treated with diaminobenzidine (DAB) reagent for color development, followed by hematoxylin counterstaining. For negative control, phosphate-buffered saline (PBS) was used instead of primary antibodies. The slices were observed under light microscopy at a magnification of × 400. Brown staining in uterus was considered as NF-κB p65 or IκB-α. The nuclear with translocation of NF-κB p65 was considered as positive. The semi-quantification of IκB-α was conducted according to a previous method with some revisions [25]. The integrated optical density (IOD) values of each slide (tissue) was obtained from its three fields with the software Image-Pro Plus 6.0. IOD index of each sample was calculated as its IOD value divided by the average IOD value of control group.

### Statistic analysis

All data are expressed as means ± standard deviation (SD). One-way analysis of variance (ANOVA) followed by Dunnett’s *t* test was employed for statistical analysis. Statistical analysis was conducted by using SPSS 16.0. Difference was considered as statistically significant when *p* < 0.05.

## Results

### APN attenuated the infiltration of inflammatory cells

**Fig. 1:**
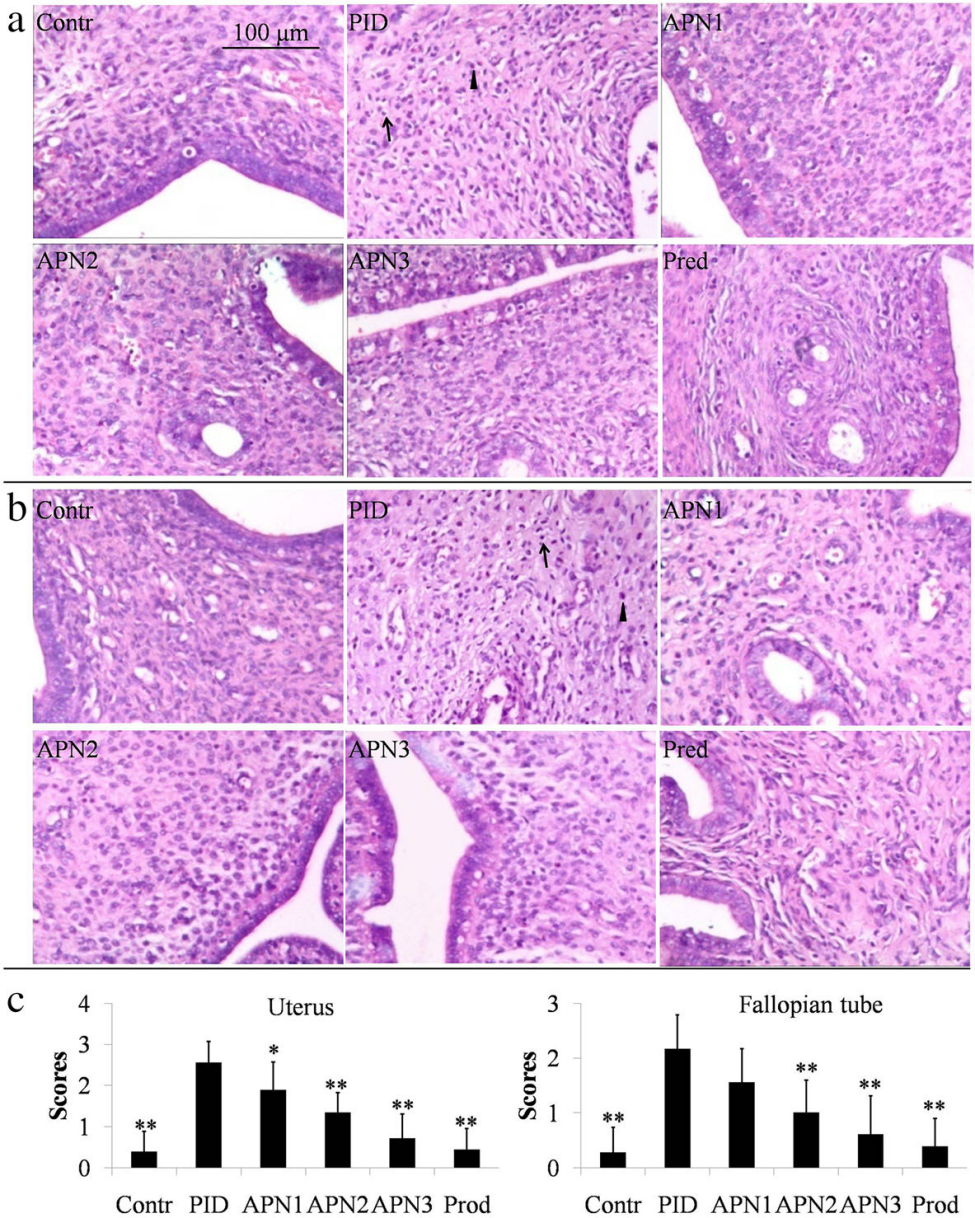
Effect of APN on pathogen-induced infiltration of neutrophils and lymphocytes in uterus and fallopian tube. Representative micrographs of uterus **a** and fallopian tube **b** stained with H & E are showed at a magnification of × 100. The infiltration of neutrophil is indicated as ▲, and the infiltration of lymphocyte is indicated as ↑. **c** Histological semi-quantitative scores of inflammatory cell. Each bar represents the mean ± SD (*P < 0.05, **P < 0.01, significantly different from PID group; *n* = 6). Contr and Pred represent control group and prednisone group, respectively

The results of histological evaluation are showed in Fig. 1. Mass inflammatory cells, including neutrophils and lymphocytes, infiltrated into the uterus and fallopian tube of the PID group rat, indicating the occurrence of inflammation in upper genital tract. After administration of APN, the infected rats showed decreased infiltrations of neutrophils and lymphocytes, compared to PID group rats. Therefore, APN could suppress the infiltration of inflammatory cells in the upper genital tract of PID rats.

### APN reduced the excessive production of cytokines and chemokines

**Fig. 2:**
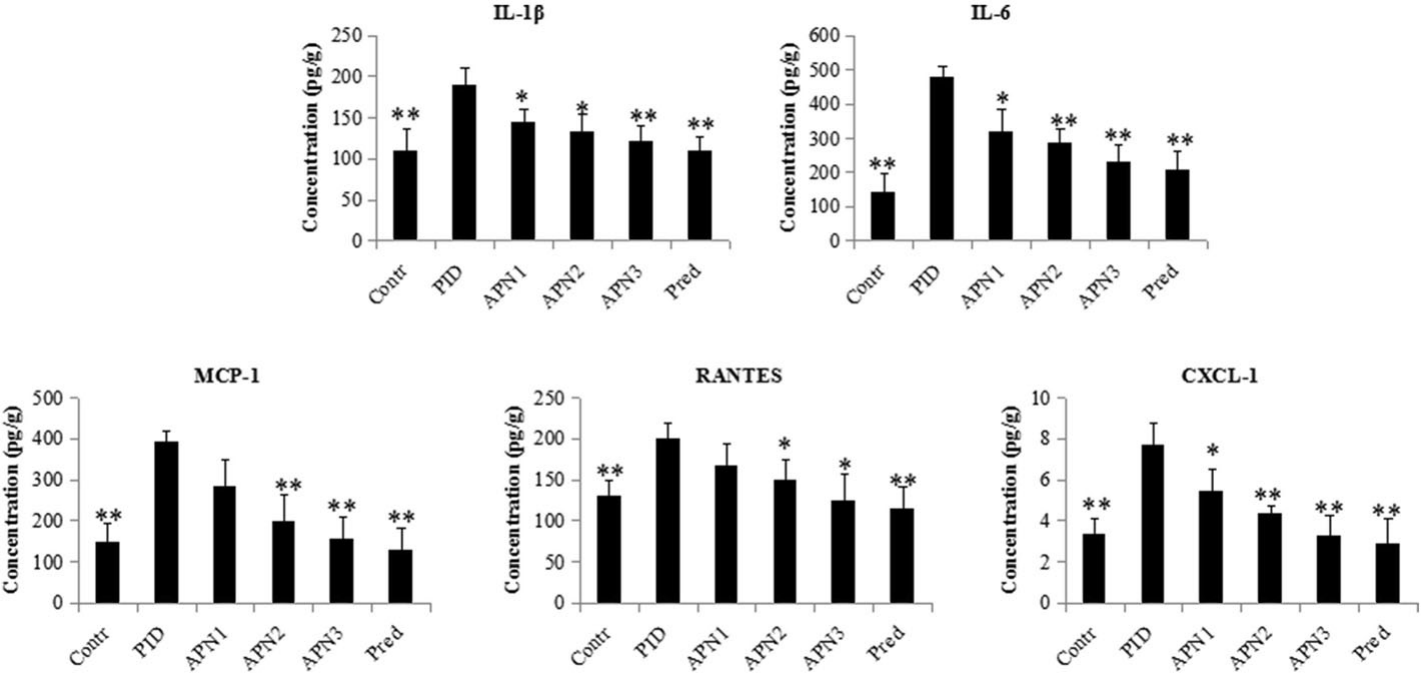
Effect of APN on pathogen-induced elevated production of IL-1β, IL-6, CXCL-1, MCP-1 and RANTES in uterus and fallopian tube. Each bar represents the mean ± SD (*P < 0.05, **P < 0.01, significantly different from PID group; *n* = 6)

To observe the inflammatory response and investigate the reason of inflammatory cells infiltration in the uterus and fallopian tube, the IL-1β, IL-6, CXCL-1, MCP-1, and RANTES in tissue homogenate were determined by using ELISA kits. As showed in Fig. 2 all these cytokines or chemokines were excessively produced in PID group compared to control group, and APN significantly reduced this pathogens-induced excessive production in a dose-dependent manner.

### APN exerted its anti-inflammatory effect via down-regulating the NF-κB pathway

**Fig. 3:**
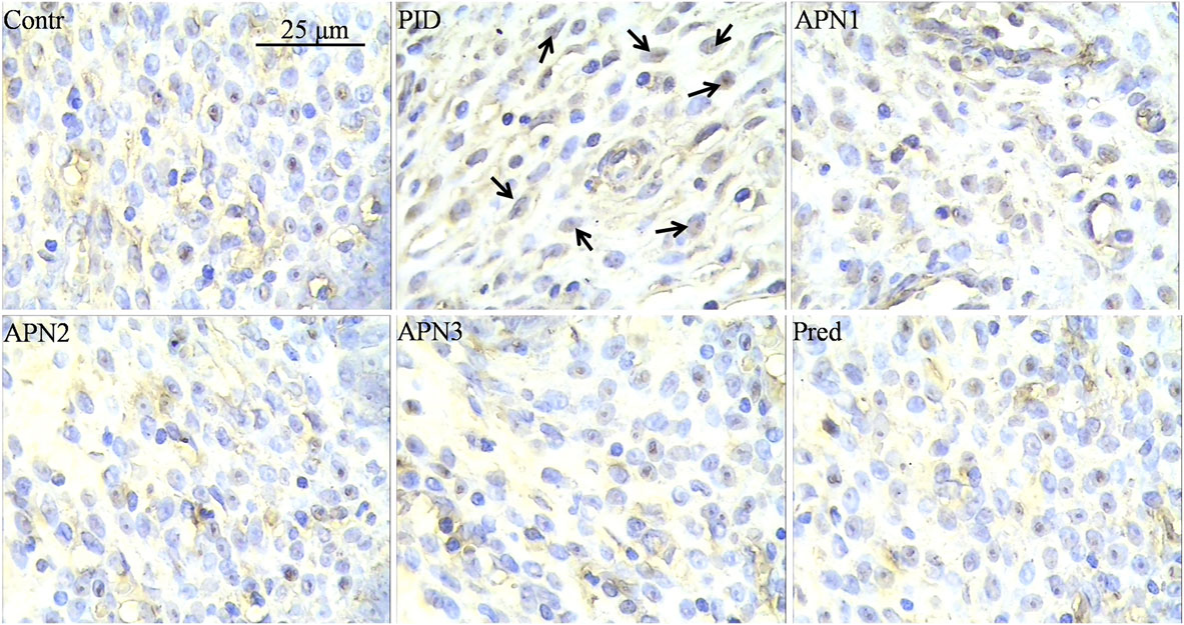
Effect of APN on pathogen-induced translocation of NF-κB p65. Representative immunohistochemistry micrographs of uterus are showed at a magnification of × 400. Arrows indicate the representative cells with nuclear translocation of NF-κB p65 (positive nuclear)

**Fig. 4:**
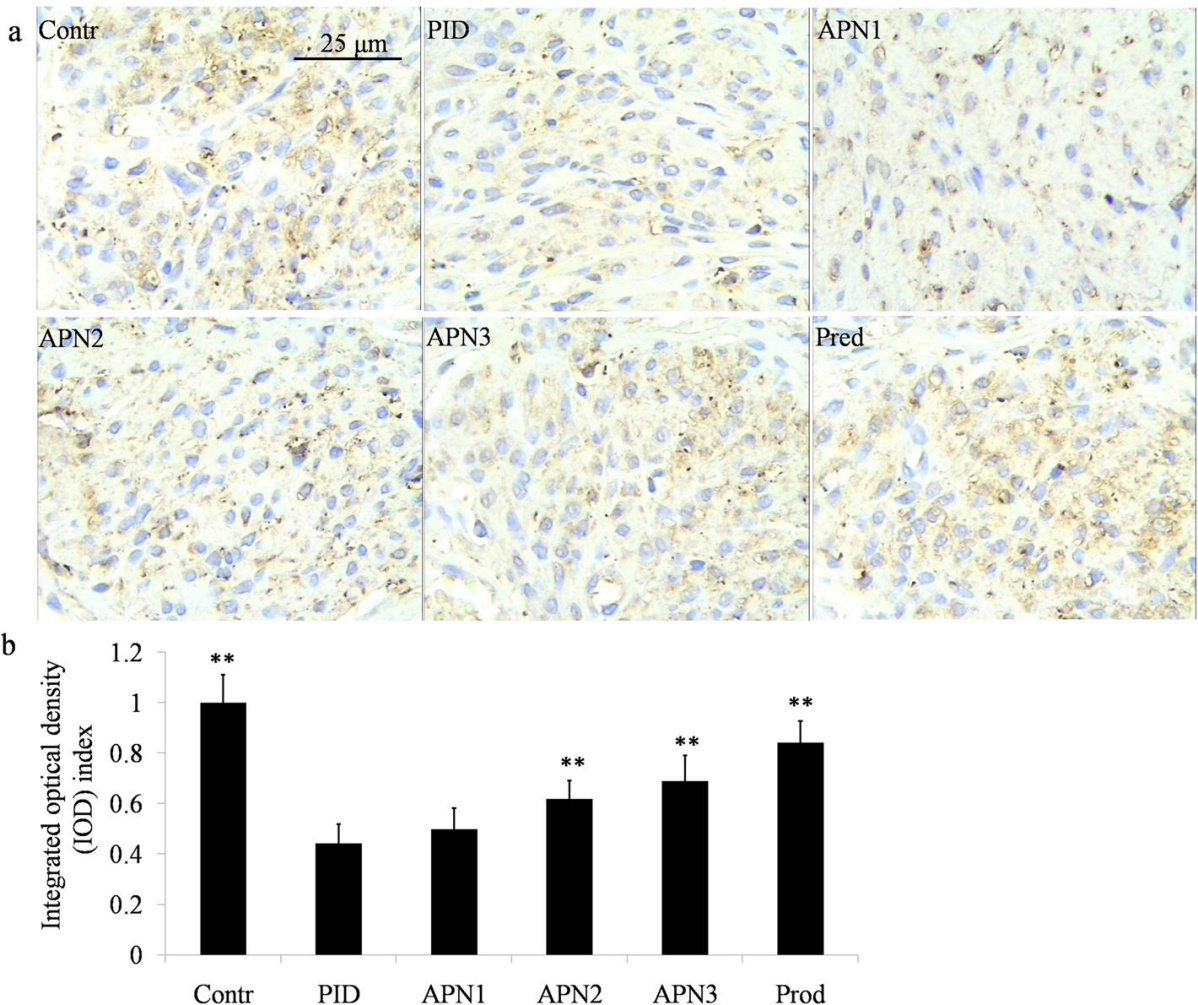
Effect of APN on pathogen-induced degradation of IκB-α. Representative immunohistochemistry micrographs of uterus **a** are showed at a magnification of × 400. **b** Semi-quantitative scores of IκB-α. Each bar represents the mean ± SD (**P < 0.01, significantly different from PID group; *n* = 6)

To illustrate a potential mechanism of anti-inflammatory effect of APN, NF-κB p65 and IκB-α, two important signaling molecules in NF-κB signaling pathway, were determined by immunohistochemical method (presented in Figs. 3 and 4, respectively). In PID group, NF-κB p65 was translocated to cell nucleus, and IκB-α showed a lower content than that in the other groups. In control, APN and prednisone groups, more NF-κB p65 was distributed in cytoplasm. These results indicated that the NF-κB pathway was up-regulated in PID group, and that APN could suppress the up-regulation of signaling pathway when the upper genital tract was infected with pathogen.

## Discussion

In clinic, the common pathogens, whose infections in upper genital tract can lead to PID, include *Chlamydia trachomatis*, *Neisseria gonorrhoeae*, genital mycoplasmas, some gram-negative and gram-positive bacteria, *etc*. [26–29]. Each pathogen could be recognized by one or more members of Toll-like receptor (TLR) family and then initiate inflammation. TLR2 and TLR4 are two important members of TLR family in upper genital tract [30], and *U. urealyticum* and *E. coli* can be recognized by TLR2 and TLR4, respectively [31, 32]. Therefore, we attempted to use *U. urealyticum* and *E. coli* mixed solution to provoke an augmented inflammation in upper genital tract.

Neutrophils in blood circulation could firmly adhere to the endothelium cell barrier, cross it, and are recruited into sites of inflammation in different pathogen-infected tissues, which is the first line of innate immune defense against pathogens [33]. Recently, study also showed the infiltration of neutrophils into the endometrium of mice with lipopolysaccharides (LPS)-induced endometritis [34]. When mass neutrophils reach the tissue at the site of infection, they will release abundant inflammatory factors, oxygen free radical and proteolytic enzyme to kill pathogens. However, the excessive products will also cause tissue damage and lead to structural disease in upper genital tract. Infiltration of lymphocyte in genital tract of mice were observed after chronic pathogen genital infection, and this infiltration play a significant role in controlling the infection [35]. Additionally, researchers found more T lymphocyte and fewer plasma cells throughout the stroma and within the epithelium [36]. Unfortunately, Patton et al. reported that the tissue damage including epithelial cell degeneration occurred close approximation to lymphocytes [37]. In the present study, after chronic pathogen infections, large number of neutrophils and lymphocytes infiltrated into the epithelium of upper genital tract, and APN showed a good activity in attenuating the infiltration of these inflammatory cells, avoiding tissue damages which is due to the inflammation.

The proinflammatory cytokines, such as IL-1β and IL-6, play essential roles on the initiation and propagation of inflammatory response, whose level in upper genital tract were increased with the pathogen infection and the recognition of immunogens by local TLRs [38–40]. At the site of inflammation, these proinflammatory cytokines stimulate the proliferation and activation of leukocyte, and enhance the production of chemokines (i.e., CXCL-1, MCP-1, and RANTES) leading to the recruitment of hematopoietic immune cells. Then, activated neutrophils will release inflammatory cytokines and chemokines, and further intensify the inflammatory response [41]. At the same time, various proinflammatory cytokines will enhance the survival or function of neutrophil [42]. In this case, the tissue damage and structural disease in upper genital tract may occur due to the intensified inflammatory response. In the present study, elevated productions of cytokines and chemokines including IL-1β, IL-6, CXCL-1, MCP-1 and RANTES were observed in upper genital tract of PID rats, indicating an obvious local inflammatory response. Besides, APN could significantly lower the levels of these cytokines and chemokines in a dose-dependent manner, suggesting a potent anti-inflammatory effect of APN for PID. As andrographolide is the major bioactive substance and has a critical effect on inhibiting the release of an important proinflammatory factor TNF-α in APN [43], it may also contribute importantly to suppressing the inflammatory response in this study.

NF-κB is a pivotal factor in promoting the transcription of genes involved in inflammatory and immune responses [44]. In most resting cells, NF-κB family members are covalently bound to IκB family members and located in the cytoplasm with no activity [45]. When the TLRs on surface of these cells recognize pathogens, resulting in the phosphorylation and degradation of IκB members, the NF-κB members will translocate to the nucleus and bind to the cis-acting NF-κB enhancer element of genes, promoting the expression of inflammatory mediators, such as IL-1β, IL-6, *etc*. [31]. These produced proinflammatory cytokines act as a positive autocrine feedback so as to the further activation of NF-κB, and subsequently more proinflammatory mediators are produced [46]. NF-κB p65 and IκB-α are representative members of NF-κB and IκB family in uterus and fallopian tube, respectively [47], and therefore were chosen as indexes to test whether the NF-κB pathway was activated in the present study. Our results demonstrated the activation of NF-κB signal pathway after multi-infection of pathogens in uterus. In the rat PID model, APN exerted its anti-inflammatory with a potential mechanism of blocking the activation of NF-κB pathway. Previous studies reporting that both of APN and its main substance andrographolide showed the effect on inhibiting NF-κB pathway in vitro support this result [48, 49]. On the other hand, andrographolide also showed the other anti-inflammatory effects, such as inhibiting JAK/STAT signaling [49], inhibiting p38 MAPK pathway [50], suppressing TLRs family expressions [49], *etc*., and whether APN have the same or more effects in upper genital tract should be verified in the further studies to facilitate its clinical use.

## Conclusions

In this study, oral administration of APN showed significant anti-inflammatory activity in pathogen-induced PID rats, including suppressing the infiltration of neutrophils and lymphocytes and reducing excessive production of cytokines or chemokines. A potential mechanism of this effect was involved in inhibiting the activation of NF-κB pathway.

## Abbreviations

APN: *Andrographis paniculata* (Burm. f.) Nees
BCA: Bicinchonininc acid
CDC: Center for disease control and prevention
DAB: Diaminobenzidine
ELISA: Enzyme-linked immunosorbent assay
H & E: Hematoxylin and eosin
IL: Interleukin
IOD: Integrated optical density
LPS: Lipopolysaccharides
NF-κB: Nuclear factor-kappa B
PBS: Phosphate-buffered saline
PID: Pelvic inflammatory disease
SD: Standard deviation
TLR: Toll-like receptor
